# Trends and characteristics of congenital syphilis in children under 1 year of age: a time series analysis, municipality of Rio de Janeiro, 2007-2021

**DOI:** 10.1590/S2237-96222026v35e20250109.en

**Published:** 2026-04-17

**Authors:** Carla Daniela Cunha Lima de Mattos, Jeane Tomazelli, Vania Reis Girianelli

**Affiliations:** 1 Secretaria Municipal de Saúde do Rio de Janeiro, Centro Municipal de Saúde Harvey Ribeiro de Souza Filho, Rio de Janeiro, RJ, Brazil; 2 Instituto Nacional de Câncer, Coordenação de Ensino, Rio de Janeiro, RJ, Brazil; 3 Fundação Oswaldo Cruz, Departamento de Direitos Humanos, Rio de Janeiro, RJ, Brazil

**Keywords:** Syphilis, Congenital, Maternal and Child Health, Race Factors, Health Information Systems, Time Series Studies, Sífilis Congénita, Salud Materno-Infantil, Factores Raciales, Sistemas de Información en Salud, Estudios de Series Temporales

## Abstract

**Objective:**

To analyze the trend and characteristics of reported cases of congenital syphilis in children under 1 year of age in the municipality of Rio de Janeiro, from 2007 to 2021.

**Methods:**

: This study employed a time series analysis using aggregated data from the Notifiable Health Conditions Information System and the Live Birth Information System. The trend in congenital syphilis rates was estimated using joinpoint regression analysis. The annual percentage change (APC) and its respective statistical significance (p-value≤0.05) were calculated for each identified segment.

**Results:**

A total of 18,544 cases of congenital syphilis were reported during the study period. The congenital syphilis rate increased from 2007 to 2012 (APC 22.2; p-value<0.001), followed by a decline until 2019 (APC -13.7; p-value 0.137), and a subsequent resurgence (APC 17.1; p-value 0.269). Black mothers had proportionally lower levels of education (12.5%), were more frequently adolescents (26.6%), had lower rates of prenatal care (86.7%), and lower rates of syphilis treatment during pregnancy (31.8%) and partner treatment (80.1%) compared to White mothers (p-value ≤0.013).

**Conclusion:**

The incidence rate of congenital syphilis in children under 1 year of age showed a marked increase until 2012, followed by a decline and a resurgence in 2020. The rate varied across planning areas and was higher among children born to Black mothers and residents of slums, although diagnosis and treatment are effective and low-cost.

## Resumen

**Objetivo:**

Analizar la tendencia y las características de los casos notificados de sífilis congénita en menores de 1 año en el municipio de Río de Janeiro en el periodo 2007-2021.

**Métodos:**

Se trata de un estudio de series temporales, utilizando datos agregados de los sistemas de información de notificación de enfermedades y nacidos vivos, estimando la tendencia de la tasa de sífilis congénita mediante el método de regresión joinpoint y evaluando la variación del porcentaje anual (APC) y su significación estadística (valor p ≤0,05) para cada segmento identificado.

**Resultados:**

Se notificaron 18 544 casos de sífilis congénita en el periodo. La tasa de sífilis congénita aumentó de 2007 a 2012 (APC 22,2; p-valor&lt;0,001), con una caída hasta 2019 (APC -13,7; p-valor 0,137) y un posterior recrudecimiento (APC 17,1; p-valor 0,269). Las madres negras tenían proporcionalmente un nivel educativo más bajo (12,5 %), eran adolescentes (26,6 %), tenían menos atención prenatal (86,7 %) y menos tratamiento para la sífilis durante el embarazo (31,8 %) y para su pareja (80,1 %) que las madres blancas (valor p ≤ 0,013).

**Conclusión:**

La tasa de incidencia de sífilis congénita en menores de 1 año de edad presentó un crecimiento significativo hasta 2012, con una caída posterior y un recrudecimiento en 2020. La tasa se presentó de forma heterogénea entre las áreas de planificación y fue mayor en niños con madres negras y residentes en favelas, a pesar de que el diagnóstico y el tratamiento son eficaces y de bajo costo.

**Ethical aspects**

This research used public-domain, anonymized databases.

## Introduction

Syphilis is the disease with the highest transmission rates during the pregnancy-puerperal cycle ^1^. Although congenital syphilis is preventable, its incidence rate remains high in Brazil ^2^, making it a persistent public health concern. 

Congenital syphilis has been considered a sentinel event for the quality of prenatal care since 1976 ^3^, but it was only included in the list of notifiable diseases ten years later ^4^. The incidence rate of congenital syphilis increased exponentially in the country, peaking in 2018 (9.0 per 1,000 live births), followed by a slight decrease over the next two years in all Brazilian regions ^2^. The global target for its control is 0.5 case of congenital syphilis per 1,000 live births ^5^. 

In 2020, the municipality of Rio de Janeiro reported twice the national incidence rate (18.4), ranking as the fourth most affected Brazilian capital ^2^. The municipality is divided into five major planning areas, which differ in terms of socioeconomic development, demographic characteristics, and the distribution and use of health services ^6^.

Several studies on congenital syphilis have been conducted in the country. A systematic review of articles published between 2007 and 2017 on the temporal and spatial distribution of congenital syphilis and associated factors included 58 studies ^7^. Of these, only one analyzed data from the municipality of Rio de Janeiro, aiming to assess prenatal care concerning vertical transmission of syphilis between 2007 and 2008 ^8^. Subsequently, a study was published on the average incidence rate of congenital syphilis for the period 2011-2014, using disaggregated data by neighborhood and planning area, but from a predictive analysis perspective ^9^. A master’s thesis was also conducted on congenital syphilis in the municipality of Rio de Janeiro during the period 2017-2019, addressing socio-spatial inequalities and access to Primary Health Care services ^10^. None of these studies analyzed trends by planning area and administrative region, nor did they cover the period of the COVID-19 pandemic.

This study aimed to analyze the trend and characteristics of reported cases of congenital syphilis in children under 1 year of age in the municipality of Rio de Janeiro between 2007 and 2021.

## Methods

Design

This was a time series study ^11^ on the incidence rate of congenital syphilis in children under 1 year of age between 2007 and 2021. 

Setting

The incidence rate of congenital syphilis in the municipality of Rio de Janeiro is among the highest in Brazilian state capitals ^2^. Despite its relevance, few studies have been conducted specifically on the municipality.

Participants

Aggregated data were used for children under 1 year of age diagnosed with congenital syphilis between 2007 and 2021 and residing in the municipality of Rio de Janeiro, as well as data on live births in the same period.

Variables

Demographic, social, and healthcare-related characteristics were analyzed, as well as the year of congenital syphilis diagnosis and the year of birth. Data on race/skin color were only assessed from 2011 onward, due to the unavailability of this variable in earlier years.

Data sources

The data were obtained from the Notifiable Health Conditions Information System and the Live Birth Information System. 

Bias control

Given the decline in the number of births, the rates were adjusted by using the average number of live births during the study period as the denominator. 

Study Size

A total of 18,544 cases of congenital syphilis recorded in the Notifiable Health Conditions Information System between 2007 and 2021 were included. A statistical power analysis was conducted considering an effect size of 0.8, a 5.0% significance level, and a two-tailed test, which revealed a power greater than 99.0%, indicating sufficient capacity to detect clinically relevant differences.

Statistical methods

The percentage of each variable category was calculated. Pearson’s chi-square test was used to assess whether statistically significant differences existed (p-value≤0.05) between White and Black race/skin color strata, with Yates’ correction applied when necessary. 

The incidence rate was calculated by dividing the number of congenital syphilis cases in children under 1 year of age by the total number of live births in the same year, multiplied by 1,000. Rates were also disaggregated by race/skin color and geographic area.

Temporal trends were described using year of notification as the independent variable and congenital syphilis incidence rate as the dependent variable. Trends were estimated using the joinpoint regression method and assessed based on annual percent change (APC). This variation indicated the direction and magnitude of the trend, as well as its statistical significance for each identified segment. 

Data were stored in Excel and analyzed using R software version 4.2.1^12^ and the Joinpoint Regression Program version 4.9.1.0 ^13^.

Data access and cleaning methods

The aggregated data were downloaded from the Rio de Janeiro municipality’s TABNET website in May 2022. 

## Results

**Figure 1.**
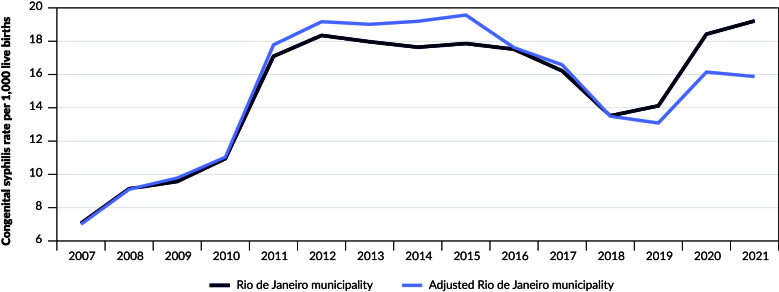
Congenital syphilis incidence rates per 1,000 live births among residents of the municipality of Rio de Janeiro, adjusted by the average number of live births. 2007-2021

A total of 18,544 cases of congenital syphilis were reported during the study period. The incidence rate of congenital syphilis in children under 1 year of age increased from 7.1 per 1,000 live births in 2007 to 19.2 in 2021 (Figure 1). Changes in the trend were identified over the period, with a marked increase from 2007 to 2012 (APC 22.2; p-value<0.001), followed by a decline through 2018, although the model smoothed the trend until 2019 (APC -13.7; p-value 0.137), and a subsequent increase (APC 17.1; p-value 0.269).

The increase in the rate from 2018 (13,5) to 2019 (14,1) was accompanied by a decline in live births in the municipality, corresponding to 82,485 and 76,576, falling to 68,265 in 2021. The adjusted rate ranged from 7.0 in 2007 to 15.9 in 2021 (Figure 1). Trend changes during the period were observed in three segments: the first extended through 2013 and remained significantly increasing (APC 20.2; p-value<0.001); the second continued until 2019 and showed a statistically significant decrease (APC -7.5; p-value 0.033); and the third showed a trend similar to the unadjusted rates (APC 0.8; p-value 0.475).

**Figure 2:**
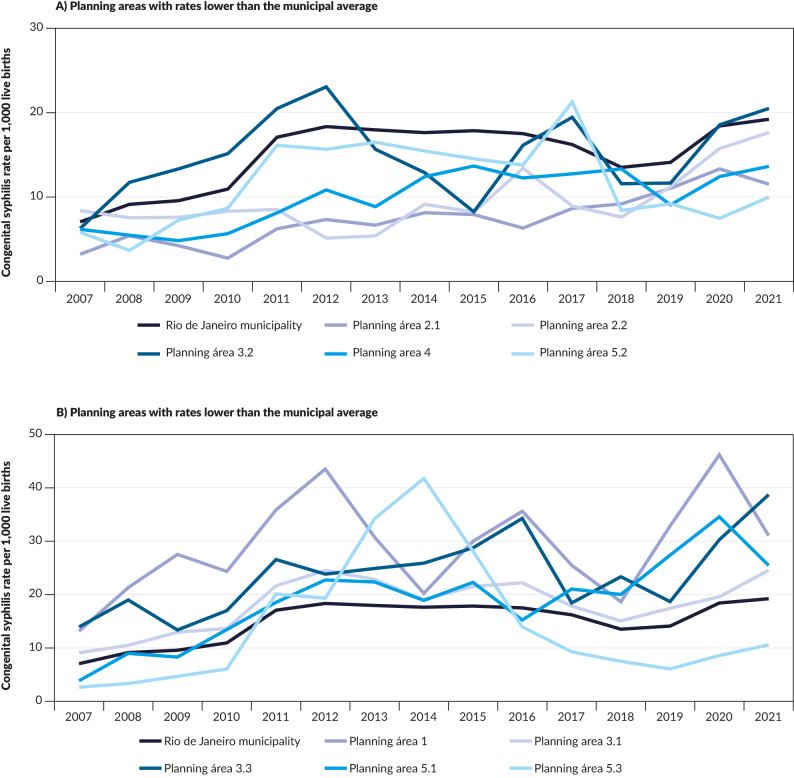
Incidence rates of congenital syphilis in children under 1 year of age, by planning area. Municipality of Rio de Janeiro, 2007-2021

**Figure 3:**
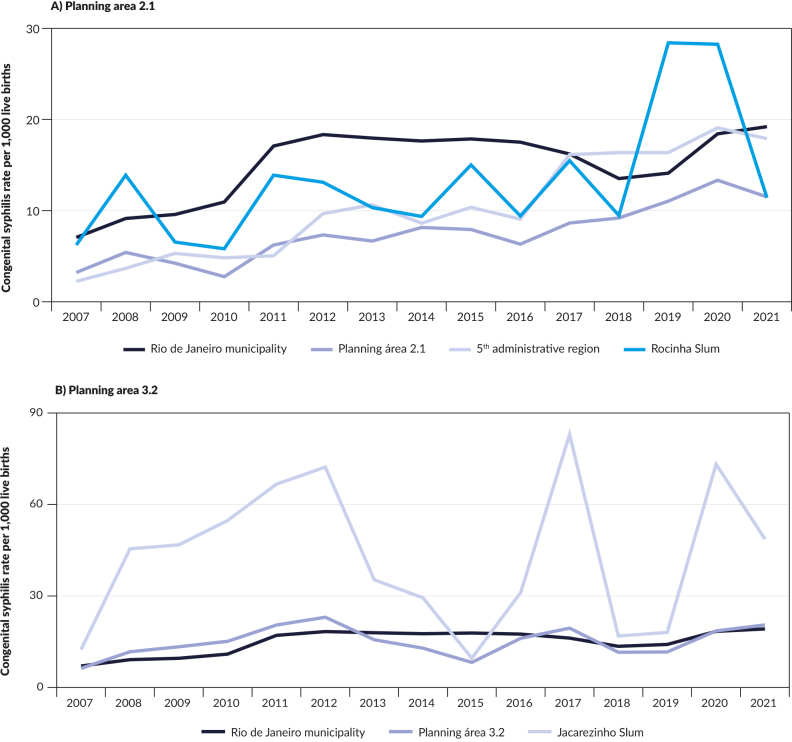
Incidence rates of congenital syphilis in children under 1 year of age, by planning areas and selected administrative regions. Municipality of Rio de Janeiro, 2007-2021

The planning areas with rates lower than those of the municipality throughout the period, but with an upward trend and an identified segment, were 2.1 (APC 9.3; p-value<0.001) and 4.0 (APC 7.0; p-value<0.001) (Figure 2A). The Copacabana (5^th^) and Rocinha (27^th^) regions had higher rates than area 2.1 (Figure 3A). The 27^th^ also exceeded the municipality’s average in 2008, 2019, and 2020, and the 5^th^ from 2018 to 2020. In Botafogo (4^th^) and Lagoa (6^th^), rates were lower than in the surrounding area, but increased in 2021. All regions showed a statistically significant upward trend (APC≥6.6; p-value≤0.024), despite fluctuations, especially in the 27^th^. 

In planning area 4.0, in the Jacarepaguá region (16^th^), rates were similar to those in the area, and in Barra da Tijuca (24^th^), they were lower, all with upward trends (APC≥5.4; p-value≤0.003). The Cidade de Deus slum (34^th^) had higher rates than the municipality as a whole from 2012 onwards, reaching more than double the rate in most years. There were three segments identified: the first from 2007 to 2009, with a statistically insignificant decline (APC -30.4; p-value 0.272), perhaps influenced by the few years of observation; the second with a significant increase (APC 40.4; p-value 0.010); and the third with stability (p-value 0.640).

Planning areas 2.2 and 5.2 had lower rates than the municipality and more than one segment (Figure 2A). Area 2.2 had a higher rate than the municipality in 2007, with an upward trend beginning in 2013 (APC 11.8; p-value 0.010). The rates in the Vila Isabel region (9^th^) were below the area average, except in 2007 and 2012; in the second segment, from 2011 onwards, the trend has risen (APC 6.8; p-value 0.034). Near Tijuca (8^th^), rates were higher than those in the area, surpassing the municipality from 2019 onwards, with an upward trend during the period (APC 6.5; p-value 0.012).

The incidence rate in area 5.2 (Figure 2A) surpassed the municipal rate in 2017, with a significant upward trend from 2007 to 2012 (APC 34.4; p-value 0.008), followed by a decline with borderline significance (APC -7.7; p-value 0.054). In Guaratiba (26^th^), rates were lower than the ones in the area for most of the period, especially after 2018, with a downward trend from 2014 onward (APC -18.7; p-value 0.047). Campo Grande (18^th^) had a lower incidence rate than those in the area until 2011, followed by an upward trend in the first segment (APC 36.7; p-value 0.003). 

Planning area 3.2 had a trend similar to that of the municipality (Figure 2A), with higher rates from 2008 to 2012, in 2017 and 2021, and an upward trend until 2011 (APC 33.3; p-value 0.003). In the Inhaúma region (12^th^), the rate was higher than that of the areas in 2013, 2017, 2019, and 2021, but remained stable across the three identified segments (p-value≥0.079). In Méier (13^th^), the rate was higher than the area’s average only in 2014, with a significant increase in incidence until 2011 (APC 39.2; p-value 0.018). In Jacarezinho slum (28^th^) (Figure 3B), rates remained above those of the municipality, except in 2015, when they more than doubled in most years, with stability in all three segments (p-value≥0.222). 

The remaining areas had rates higher than the municipal average (Figure 2B). In area 1.0, a single segment with overall stability was identified (p-value 0.106), despite substantial fluctuations. The Centro (2^nd^) and Rio Comprido (3^rd^) regions had one segment with higher rates than those in the area starting in 2014, exhibiting upward trends (APC≥6.7; p-value≤0.006). In the other regions, three segments were identified. The Port Region (1^st^) experienced a sharp increase until 2011 (APC 40.6; p-value 0.046), with rates below the area’s average except in 2010, and stability in subsequent segments (p-value ≥ 0.103). In São Cristóvão (7^th^), rates were higher than those of the area until 2013, but stable (p-value 0.070), followed by a significant decline through 2019 (APC -18.5; p-value 0.026) and subsequent stability (p-value 0.293).

In planning area 3.1, three segments were identified, with a statistically significant increase only until 2012 (APC 22.6; p-value 0.001). The Ilha do Governador (20^th^) and Maré slum (30^th^) regions had rates below the area’s average in most years. In the 30^th^ region, there was one segment with an upward trend over the period (APC 9.9; p-value<0.001). In the 20^th^ region, three segments were identified, with a sharp upward trend until 2012 (APC 44.3; p-value 0.012). The Complexo do Alemão slum (29^th^) experienced several years without notification, which made analysis impossible. In the other regions, three segments were also identified. In Ramos (10^th^), rates exceeded those of the area throughout the period, with a sharp increase until 2012 (APC 35.9; p-value 0.001), followed by a decline until 2019 (APC -10.8; p-value 0.002) and later stability (p-value 0.934). In Penha (11^th^), rates exceeded those of the area until 2012, with an upward trend (APC 20.2; p-value<0.001), and again after 2019 (APC 35.9; p-value 0.005). However, rates showed a sharp decline from 2012 to 2019 (APC -20.4; p-value 0.002). In Vigário Geral (31^st^), rates exceeded those of the area until 2016, with an upward trend (APC 11.2; p-value 0.003). There was a borderline decline until 2019 (APC -46.6; p-value 0.052), followed by a subsequent significant growth (APC 132.7; p-value 0.016).

In planning area 3.3, three segments were identified, with a statistically significant upward trend only in the first (2007-2015) (APC 9.8; p-value 0.022). All of its regions also had three segments. In Irajá (14^th^) and Anchieta (22^nd^), rates were lower than those of the municipality throughout the period. In the 14^th^ region, the trend was upward until 2016 (APC 8.1; p-value 0.032), followed by stability until 2019 (p-value 0.323) and a sharp increase thereafter (APC 117.4; p-value 0.046). In the 22^nd^ region, a sharp increase occurred until 2011 (APC 28.4; p-value 0.034), followed by stability in later segments (p-value≥0.524). Rates in Madureira (15^th^) surpassed those of the area beginning in 2019, but the upward trend only occurred until 2011, with borderline significance (APC 24.0; p-value 0.056). In Pavuna (25^th^), rates exceeded those of the area in 2007 and from 2014 to 2017, with an upward trend until 2016 (APC 12.0; p-value 0.018). 

In planning area 5.1, rates exceeded those of the municipality from 2010 onward, with an upward trend until 2012 (APC 38.5; p-value 0.001). In Bangu (17^th^), rates were higher than in the area except in 2018, with an upward trend until 2012 (APC 39.3; p-value 0.001). In contrast, in Realengo (33^rd^), rates were lower than those in the area except in 2018, with an upward trend until 2011 (APC 41.5; p-value 0.035). Rates in planning area 5.3 exceeded those of the municipality between 2011 and 2015, with an upward trend until 2011 (APC 41.5; p-value 0.035).

Data on race/skin color showed a high percentage of unknown information in congenital syphilis notifications on the Notifiable Diseases Information System. The lowest percentage was noted in 2017 (24.2%) and the highest in 2011 (48.2%). The Live Birth Information System showed a high percentage in 2011 (36.4%) and varied from 1.0% in 2013 to 3.5% in 2021.

Black mothers had proportionally lower schooling and age (p-value 0.004) than white mothers (Table 1). They also had less prenatal care (p-value<0.001) and treatment for syphilis during pregnancy (p-value<0.001) and in their partner (p-value 0.013). They also had lower rates of prenatal care (p-value<0.001), syphilis treatment during pregnancy (p-value<0.001), and partner treatment (p-value 0.013). A high proportion of cases involved mothers who attended prenatal care-both White and Black-who were diagnosed during pregnancy but received inadequate maternal treatment. 

**Figure 4:**
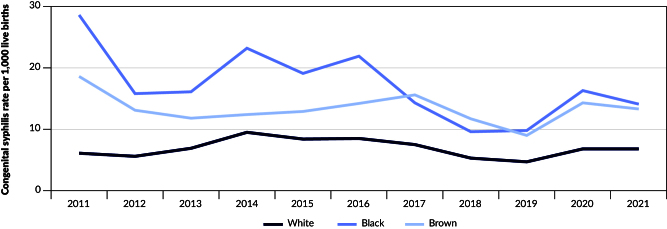
Incidence rates of congenital syphilis in children under 1 year of age, according to race/skin color. Municipality of Rio de Janeiro, 2007-2021

Rates among White mothers were the lowest (Figure 4), peaking in 2014 but remaining stable over the period (p-value 0.647). Rates among Brown-skinned mothers were higher than among White mothers, peaking in 2017; however, the observed downward trend was not statistically significant (APC -1.9; p-value 0.306). The highest rates were found among Black mothers, although they were lower than those of Brown-skinned mothers in 2017 and 2018; this group showed a significant downward trend over the period (APC -6.1; p-value 0.040).

**Table 1:** Characteristics of congenital syphilis cases by maternal race/skin color. Municipality of Rio de Janeiro, 2011-2021 (n=9,693)

Sociodemographic characteristics	White	Black	p-value
	n (%)	n (%)	
Maternal age group (years)			0.004
out/19	549 ( 24.4)	1,929 ( 26.6)	
20+	1,703 ( 75.6)	5,326 ( 73.4)	
Unknown	41 ( 1.8)	145 ( 2.0)	-
Maternal educational level			0.004
Until the 4^th^ grade of elementary school	172 ( 9.9)	672 ( 12.5)	
5^th^ grade or higher	1,566 ( 90.1)	4,711 ( 87.5)	
Unknown	555 ( 24.2)	2,017 ( 27.3)	-
Prenatal care			<0.001
Yes	2,020 ( 90.8)	6,171 ( 86.7)	
No	204 ( 9.2)	950 ( 13.3)	
Unknown	69 ( 3.0)	279 ( 3.8)	-
When the diagnosis of maternal syphilis was made			0.195
During prenatal care	1,347 ( 61.0)	4,250 ( 59.5)	
At the time of delivery/curettage	787 ( 35.6)	2,602 ( 36.5)	
After delivery	70 ( 3.2)	243 ( 3.4)	
Not performed	6 ( 0.3)	43 ( 0.6)	
Unknown	83 ( 3.6)	262 ( 3.5)	-
Maternal treatment regimen			<0.001
Appropriate	96 ( 4.7)	333 ( 5.1)	
Inappropriate	1398	4,110 ( 63.1)	
	(68.7)		
Not performed	541	2,072 ( 31.8)	
	(26.6)		
Unknown	258 ( 11.3)	885 ( 12.0)	-
Intimate partner treatment			0.013
Yes	327 ( 23.0)	870 ( 19.9)	
No	1,094 ( 77.0)	3,504 ( 80.1)	
Unknown	872 ( 38.0)	3,026 ( 40.9)	-
Total	2,293 ( 100.0)	7,400 ( 100.0)	-

## Discussion

The incidence rate of congenital syphilis in children under 1 year of age showed a marked increase from 2007 to 2012, followed by a decline until 2019, although not statistically significant. The rate varied across planning areas and was higher among children born to Black and Brown mothers. 

The main limitation of this study was the incompleteness of the race/skin color variable in the database, which hindered a more comprehensive understanding of its influence on the trend of congenital syphilis. Studies conducted in Recife ^14^, Belo Horizonte ^15^, and the city of Rio de Janeiro ^9^ found that children born to Black mothers with low levels of education had an increased risk of congenital syphilis. Another limitation was the fluctuations in rates, most likely random, in some areas. The moving average method was not used, as aggregating data by period could mask the impact of public policies. The Joinpoint Regression method was applied instead, because it smooths the curve to estimate segmented trends. Underreporting may have affected the results and is more related to the type of health facility than to individual characteristics ^16^. A study assessing fetal and infant deaths from congenital syphilis identified 0.9% underreporting in the Notifiable Health Conditions Information System and 7.0% in the Mortality Information System ^17^.

The decline in congenital syphilis incidence coincided with the implementation of the Rede Cegonha program. This policy focused on humanized care within the public health system, emphasizing the role of nurse-midwives and midwives in maternal care ^18^. The shortage of benzathine penicillin, the antibiotic used to treat syphilis in pregnant women and their infants, may have minimized the impact of declining rates between 2014 and 2017 ^19^. Starting in 2018, the disease resurged, shortly after the dismantling of Primary Health Care in the municipality of Rio de Janeiro ^20^, and was further aggravated by the COVID-19 pandemic. The worsening of congenital syphilis was not statistically significant, likely due to the short follow-up period. 

The effect of implementing a policy to enhance prenatal care was slow, with only a slight downward trend in congenital syphilis rates. By contrast, the dismantling of services had an immediate impact, resulting in an exponential upward trend. These hypotheses could not be statistically confirmed and were not observed at the national or macroregional levels, where the trend remained upward from 2011 to 2021 ^21^. The sharp decline in live births from 2018 onward does not explain the increase in congenital syphilis incidence, as the adjusted trend-based on the average number of live births-was similar to that of the unadjusted rates. 

In 2022, the Rede Cegonha was replaced nationwide by the Maternal and Child Care Network ^22^ ^,^ ^23^. This change may negatively affect maternal care, as the new network prioritizes a hospital-centered, physician-centered model over the territory-based care model previously conducted by the Family Health Strategy. It is also disconnected from the national reality and the principles of the Brazilian National Health System ^24^. In 2023, this network was deactivated ^25^, and its impact is still unknown.

The incidence of congenital syphilis was not homogeneous across the municipality. Planning areas 1.0, 3.3, and 5.1 had consistently higher rates than the municipal average. In Area 2.1, located in the South Zone, despite lower overall rates, the trend was upward-likely influenced by densely populated and underserved neighborhoods, such as the Rocinha slum, where rates surpassed the municipal average. A similar situation was observed in area 4.0, where rates were lower in Barra da Tijuca but much higher in the Cidade de Deus slum. Jacarezinho slum also showed high rates of congenital syphilis throughout the period, diverging from other regions in area 3.2. Only in Guaratiba and Complexo do Alemão slum, no increases in rates were observed from 2018 onwards. The rates for Complexo do Alemão slum were below those for its area (3.1) and the municipality. Studies in Recife ^14^ and the municipality of Rio de Janeiro ^9^ ^,^ ^10^ also identified heterogeneous distribution across neighborhoods, with higher congenital syphilis rates in areas with worse socioeconomic and health care indicators. 

The supply of penicillin was uneven across planning areas, with greater shortages in areas 3.0 and 5.0, particularly in 2014, 2015, and 2017 ^19^. However, this situation was not reflected in the trend of congenital syphilis incidence in these areas. Only area 3.3 showed a resurgence in the rate as of 2013; however, a decline began in 2016, and it did not influence the change in the trend, as it had already been high and rising since 2007. 

Most mothers of children with congenital syphilis underwent prenatal screening and were diagnosed with syphilis, but treatment was inadequate or not provided-a situation similar to national data ^21^. Despite efforts, syphilis remains a serious public health problem due to failures in prenatal care ^26^. High turnover among Primary Health Care professionals-due to outsourcing contracts-may contribute to this issue, as replacements are often not accompanied by training, compromising the quality of care provided.

The proportion of missing race/skin color data in the Notifiable Health Conditions Information System remained high, even after 2017 when filling out this field became mandatory across all health information systems ^27^, and was more than double the national average throughout the study period ^21^. When missing or unknown data exceed 10.0%, analysis is compromised, as the data may not be randomly distributed across categories ^28^. Given this situation, the interpretation of race/skin color data must be approached with caution. 

In this study, Black mothers had lower prenatal care rates than White mothers. The incidence rates of congenital syphilis in children born to Black and Brown mothers were higher than among those born to White mothers. However, all groups showed a downward trend until 2019, followed by a resurgence. Inequality in access to prenatal care and diagnostic testing by maternal race/skin color had already been identified in the National Health Survey ^29^ and international literature ^30^.

Despite the availability of effective and low-cost diagnostic and treatment methods, efforts to combat congenital syphilis in the municipality of Rio de Janeiro have not achieved the desired outcomes, particularly among the most vulnerable groups, such as Black populations and residents of slums. The dismantling of Primary Health Care and the instability of employment contracts are among the main obstacles. The abolition of the Maternal and Child Care Network represents progress in reversing this regression, but it is not enough. 

It is necessary to establish a human resources policy for the Brazilian National Health System, especially for Primary Health Care, to ensure workforce retention, which would lead to lower training costs and improved effectiveness of care delivery. Moreover, investment is needed in training for qualified listening, to strengthen bonds and foster research aimed at identifying tailored strategies for care, promoting greater treatment adherence among pregnant women and their partners.

## Data Availability

Following the Open Science Compliance form, the content underlying the manuscript will be made available to the authors upon request after publication.
