# Supplementary material for: Trends and characteristics of congenital syphilis in children under 1 year of age: a time series analysis, municipality of Rio de Janeiro, 2007-2021
Source: Epidemiol Serv Saude. 2026 Apr 17;35:e20250109. doi: 10.1590/S2237-96222026v35e20250109.en (PMC13090015; doi:10.1590/S2237-96222026v35e20250109.en)
Supplement: Translation

# Translation: Tendências e características da sífilis congênita em menores de 1 ano de idade: análise de série temporal, município do Rio de Janeiro, 2007-2021

**DOI:** 10.1590/S2237-96222026v35e20250109.pt

**Authors:** Carla Daniela Cunha Lima de Mattos, Jeane Tomazelli, Vania Reis Girianelli

**Affiliations:** 1 Secretaria Municipal de Saúde do Rio de Janeiro, Centro Municipal de Saúde Harvey Ribeiro de Souza Filho, Rio de Janeiro, RJ, Brasil; 2 Instituto Nacional de Câncer, Coordenação de Ensino, Rio de Janeiro, RJ, Brasil; 3 Fundação Oswaldo Cruz, Departamento de Direitos Humanos, Rio de Janeiro, RJ, Brasil

**Keywords:** Sífilis Congênita, Saúde Materno-infantil, Fatores Raciais, Sistemas de Informação em Saúde, Estudos de Séries Temporais

## Abstract

**Objetivo:**

: Analisar a tendência e as características dos casos notificados de sífilis congênita em menores de 1 ano de idade no município do Rio de Janeiro no período 2007-2021.

**Métodos:**

: Tratou-se de estudo de séries temporais, utilizando dados agregados dos sistemas de informação de agravos de notificação e nascidos vivos, sendo estimada a tendência da taxa de sífilis congênita pelo método de regressão *joinpoint* e avaliada a variação do percentual anual (APC) e respectiva significância estatística (p-valor ≤0,005) para cada segmento identificado.

**Resultados:**

: Foram notificados 18.544 casos de sífilis congênita no período. A taxa de sífilis congênita aumentou de 2007 a 2012 (APC 22,2; p-valor<0,001), com queda até 2019 (APC -13,7; p-valor 0,137) e posterior recrudescimento (APC 17,1; p-valor 0,269). As mães negras tinham proporcionalmente escolaridade mais baixa (12,5%), eram adolescentes (26,6%), menor realização de pré-natal (86,7%) e tratamento para sífilis na gestação (31,8%) e do parceiro (80,1%) do que as mães brancas (p-valor≤0,013).

**Conclusão:**

: A taxa de incidência de sífilis congênita em menores de 1 ano de idade apresentou crescimento expressivo até 2012, com queda posterior e recrudescimento em 2020. A taxa ocorreu de forma heterogênea entre as áreas de planejamento e foi maior em crianças com mães negras e residentes em favela, apesar de o diagnóstico e o tratamento serem efetivos e de baixo custo.

**Aspectos éticos**

Esta pesquisa utilizou bancos de dados de domínio público e anonimizados.

## Introdução

A sífilis é a doença que apresenta as maiores taxas de transmissão durante o ciclo gravídico-puerperal ^1^. A sífilis congênita pode ser evitada, mas a taxa de incidência ainda permanece alta no Brasil ^2^, mantendo-a como relevante problema de saúde pública. 

A sífilis congênita é considerada evento sentinela da qualidade da assistência pré-natal desde 1976 ^3^, mas só após dez anos foi incluída na lista de notificação compulsória ^4^. A taxa de incidência de sífilis congênita aumentou exponencialmente no país, com pico em 2018 (9,0/1 mil nascidos-vivos) e ligeira queda nos dois anos posteriores em todas as regiões brasileiras ^2^. A meta mundial para o seu controle é 0,5 caso de sífilis congênita por 1 mil nascidos vivos ^5^. 

Em 2020, o município do Rio de Janeiro apresentou o dobro da taxa de incidência do país ^18^ ^,^ ^4^, sendo a quarta capital brasileira mais atingida ^2^. O município está dividido em cinco grandes áreas de planejamento que apresentam diferenças quanto ao desenvolvimento socioeconômico e às características demográficas, bem como na distribuição e utilização dos serviços de saúde ^6^.

Diversos estudos sobre sífilis congênita têm sido realizados no país. Em revisão sistemática dos artigos publicados de 2007 a 2017, sobre a distribuição temporal e espacial da sífilis congênita e os fatores associados, foram incluídos 58 artigos ^7^. Destes, apenas um analisava os dados do município do Rio de Janeiro e com objetivo de avaliar a assistência pré-natal na transmissão vertical da sífilis entre 2007 e 2008 ^8^. Posteriormente, foi publicado um estudo sobre a taxa de incidência média de sífilis congênita para o período 2011-2014, com dados desagregados por bairros e respectivas áreas de planejamento, mas sob a perspectiva da análise preditiva ^9^. Também foi realizada uma dissertação sobre sífilis congênita no município do Rio de Janeiro, no período 2017-2019, que abordou as desigualdades socioespaciais e de acesso aos serviços de Atenção Primária à Saúde ^10^. Nenhum dos estudos abordou a tendência por área de planejamento e região administrativa e contemplou o período da pandemia de covid-19.

Este estudo teve como objetivo analisar a tendência e as características dos casos notificados de sífilis congênita em menores de 1 ano de idade no município do Rio de Janeiro entre 2007 e 2021.

## Métodos

Delineamento

Tratou-se de estudo de séries temporais ^11^ ^)^ da taxa de incidência de sífilis congênita em menores de 1 ano de idade entre 2007 e 2021. 

Contexto

A taxa de incidência de sífilis congênita do município do Rio de Janeiro é uma das maiores entre as capitais brasileiras ^2^. Apesar da relevância, identificou-se carência de estudos para o município.

Participantes

Foram utilizados os dados agregados de crianças menores de 1 ano de idade com diagnóstico de sífilis congênita entre 2007 e 2021 e residentes no município do Rio de Janeiro, bem como os dados dos nascidos vivos no mesmo período.

Variáveis

Utilizaram-se as características demográficas, sociais e assistenciais, o ano de diagnóstico da sífilis congênita e o ano de nascimento. Os dados referentes à raça/cor da pele só foram avaliados a partir de 2011, devido à indisponibilidade da variável no período anterior.

Fonte de dados

Foram considerados os casos do Sistema de Informação de Agravos de Notificação e a população do Sistema de Informações sobre Nascidos Vivos. 

Controle de viés

Tendo em vista a queda nos nascimentos, as taxas foram ajustadas no denominador para média de nascidos-vivos no período. 

Tamanho do estudo

Foram incluídos os 18.544 casos de sífilis congênita registrados no Sistema de Informação de Agravos de Notificação entre 2007 e 2021. Realizou-se análise de poder estatístico considerando o tamanho de efeito de 0,8, o nível de significância de 5% e o teste bilateral, que revelou poder superior a 99%, indicando ser suficiente para detectar diferenças clinicamente relevantes.

Métodos estatísticos

O percentual de cada categoria das variáveis estudadas foi calculado. O teste qui-quadrado de Pearson utilizado para avaliar a existência de diferença estatisticamente significativa (p-valor≤0,05) entre os estratos de raça/cor da pele branca e negra, com correção de Yates se necessário. 

A taxa de incidência foi calculada dividindo o número de casos de sífilis congênita em menores de 1 ano de idade pelo total de nascidos-vivos, por ano de diagnóstico, vezes 1 mil. As taxas também foram desagregadas por raça/cor da pele e áreas.

A tendência temporal foi descrita tendo, como variável independente, o ano da notificação e, como variável dependente, a taxa de incidência de sífilis congênita, sendo estimada pelo método de regressão *joinpoint* e avaliada pela variação do percentual anual (*annual percent change* - APC). Essa variação sinalizou a direção, magnitude da tendência e respectiva significância estatística para cada segmento identificado. 

Os dados foram armazenados no Excel e analisados nos programas R versão 4.2.1 ^12^ e Joinpoint Regression Program versão 4.9.1.0 ^13^.

Acesso aos dados e métodos de limpeza

Realizou-se download dos dados agregados disponibilizados na página eletrônica do TABNET do município do Rio de Janeiro em maio de 2022. 

## Resultados

Foram notificados 18.544 casos de sífilis congênita no período. A taxa de incidência de sífilis congênita em menores de 1 ano de idade aumentou de 7,1 por 1 mil nascidos vivos em 2007 para 19,2 em 2021 (Figura 1). Mudanças na tendência durante o período foram identificadas com crescimento expressivo de 2007 a 2012 (APC 22,2; p-valor<0,001), queda até 2018, mas com alisamento da tendência pelo modelo até 2019 (APC -13,7; p-valor 0,137), e posterior incremento (APC 17,1; p-valor 0,269).

O aumento da taxa de 2018 (13,5) a 2019 (14,1) foi acompanhada da queda de nascidos vivos no município, correspondendo a 82.485 e 76.576 e atingindo 68.265 em 2021. A taxa ajustada variou de 7,0 em 2007 a 15,9 em 2021 (Figura 1). Mudanças na tendência no período foram observadas em três segmentos: o primeiro se prolongou até 2013 e permaneceu com aumento expressivo (APC 20,2; p-valor<0,001); o segundo se estendeu até 2019 e passou a ter queda estatisticamente significativa (APC -7,5; p-valor 0,033); e o terceiro segmento apresentou tendência similar às taxas não ajustadas (APC 0,8; p-valor 0,475).

As áreas de planejamento com taxas inferiores às do município ao longo do período, mas com tendência crescente e um segmento identificado, foram: 2.1 (APC 9,3; p-valor<0,001) e 4.0 (APC 7,0; p-valor<0,001) (Figura 2A). As regiões Copacabana (5ª) e Rocinha (27ª) apresentaram taxas superiores à da área 2.1 (Figura 3A). A 27ª superou também o município em 2008, 2019 e 2020; e a 5ª, de 2018 a 2020. Nas regiões Botafogo (4ª) e Lagoa (6ª), as taxas estavam abaixo da área, sendo maiores em 2021. Todas as regiões tiveram tendência crescente estatisticamente significativa (APC≥6,6; p-valor≤0,024), apesar de flutuações, principalmente a 27ª. 

**Figura 1.** Taxas de incidência de sífilis congênita por 1 mil nascidos vivos de residentes no município do Rio de Janeiro e ajustadas pela média de nascidos vivos. 2007-2021

Na área de planejamento 4.0, na região de Jacarepaguá (16ª), as taxas foram similares à da área, e, na Barra da Tijuca (24ª), inferiores, todas com tendência de crescimento no período (APC≥5,4; p-valor≤0,003). A favela Cidade de Deus (34ª) apresentou taxas superiores às do município a partir de 2012 e atingiu mais que o dobro na maioria dos anos, sendo identificados três segmentos: o primeiro de 2007 a 2009, com declínio não estatisticamente significativo (APC -30,4; p-valor 0,272), talvez influenciado pelos poucos anos de observação; o segundo com aumento expressivo (APC 40,4; p-valor 0,010); e o terceiro com estabilidade (p-valor 0,640).

Nas áreas de planejamento 2.2 e 5.2, as taxas foram mais baixas que à do município e com mais de um segmento (Figura 2A). A área 2.2 apresentou taxa mais elevada do que à do município em 2007, com tendência crescente a partir de 2013 (APC 11,8; p-valor 0,010). As taxas da região de Vila Isabel (9ª) ficaram abaixo da área, exceto em 2007 e 2012; no segundo segmento, com início em 2011, a tendência foi crescente (APC 6,8; p-valor 0,034). Na Tijuca (8ª), as taxas foram mais elevadas do que às da área, superando o município a partir de 2019, com tendência de crescimento no período (APC 6,5; p-valor 0,012).

A taxa da área de planejamento 5.2 (Figura 2A) superou à do município em 2017, com tendência crescente no período 2007-2012 (APC 34,4; p-valor 0,008) e queda posterior, com significância estatística limítrofe (APC -7,7; p-valor 0,054). Na região de Guaratiba (26ª), as taxas estavam abaixo da área, na maior parte do período observado, principalmente a partir de 2018, e com tendência decrescente a partir de 2014 (APC -18,7; p-valor 0,047). Campo Grande (18ª) apresentou taxa de incidência abaixo da área até 2011, com tendência crescente no primeiro segmento (APC 36,7; p-valor 0,003). 

A área de planejamento 3.2 apresentou tendência similar ao município (Figura 2A), com taxas superiores no período 2008-2012, em 2017 e em 2021, com tendência crescente até 2011 (APC 33,3; p-valor 0,003). Na região de Inhaúma (12ª), a taxa foi superior à da área em 2013, 2017, 2019 e 2021, mas com estabilidade nos três segmentos identificados (p-valor≥0,079). No Méier (13ª), a taxa foi superior à área apenas em 2014, com expressivo aumento da incidência até 2011 (APC 39,2; p-valor 0,018). Na favela do Jacarezinho (28ª) (Figura 3B), as taxas mantiveram-se acima das do município, exceto em 2015, atingindo mais que o dobro na maioria dos anos, e com estabilidade nos três segmentos (p-valor≥0,222). 

As demais áreas apresentaram taxas superiores às do município (Figura 2B). Na área de planejamento 1.0, foi identificado um segmento com estabilidade no período (p-valor 0,106), mas com grande flutuação. As regiões Centro (2ª) e Rio Comprido (3ª) apresentaram um segmento e taxas superiores às da área a partir de 2014, mas com tendência crescente no período (APC≥6,7; p-valor≤0,006). Nas demais, foram identificados três segmentos. A região Portuária (1ª) apresentou aumento expressivo até 2011 (APC 40,6; p-valor 0,046), com taxas abaixo da área, exceto em 2010, e estabilidade nos segmentos posteriores (p-valor≥0,103). Em São Cristóvão (7ª), as taxas foram superiores à da área até 2013, mas com estabilidade (p-valor 0,070), e houve declínio expressivo até 2019 (APC 18,5; p-valor 0,026), com posterior estabilidade (p-valor 0,293).

**Figura 2.** Taxas de incidência de sífilis congênita em menores de 1 ano de idade, por área de planejamento. Município do Rio de Janeiro, 2007-2021

**Figura 3.** Taxas de incidência de sífilis congênita em menores de 1 ano de idade, por áreas de planejamento e respectivas regiões administrativas selecionadas. Município do Rio de Janeiro, 2007-2021

Na área de planejamento 3.1, foram identificados três segmentos, com crescimento estatisticamente significativo somente até 2012 (APC 22,6; p-valor 0,001). As regiões Ilha do Governador (20ª) e favela da Maré (30ª) apresentaram taxas abaixo da área, na maioria dos anos. Na 30ª, foi identificado um segmento e tendência de crescimento no período (APC 9,9; p-valor<0,001). Na 20ª, foram identificados três segmentos, mas com tendência de aumento até 2012 (APC 44,3; p-valor 0,012), embora expressiva. A favela Complexo do Alemão (29ª) teve vários anos sem notificação, impossibilitando a análise. Nas demais, também foram identificados três segmentos. Em Ramos (10ª), as taxas superaram às da área em todo o período, com incremento importante até 2012 (APC 35,9; p-valor 0,001), declínio até 2019 (APC -10,8; p-valor 0,002) e estabilidade posterior (p-valor 0,934). A Penha (11ª) apresentou taxas superiores às da área até 2012 com tendência crescente (APC 20,2; p-valor<0,001) e após 2019 (APC 35,9; p-valor 0,005), com declínio acentuado no período 2012-2019 (APC -20,4; p-valor 0,002). Em Vigário Geral (31ª), as taxas superaram às da área até 2016, com tendência de aumento (APC 11,2; p-valor 0,003), declínio até 2019, embora limítrofe (APC -46,6; p-valor 0,052), e crescimento posterior expressivo (APC 132,7; p-valor 0,016).

Na área de planejamento 3.3, foram identificados três segmentos no período, mas apenas o primeiro (2007-2015) com tendência de crescimento estatisticamente significativa (APC 9,8; p-valor 0,022). Também foram identificados três segmentos em todas as suas regiões. Em Irajá (14ª) e Anchieta (22ª), as taxas foram inferiores às do município em todo o período avaliado. Na 14ª, a tendência foi crescente até 2016 (APC 8,1; p-valor 0,032), seguida de estabilidade até 2019 (p-valor 0,323) e incremento expressivo posteriormente (APC 117,4; p-valor 0,046). Na 22ª, a tendência de aumento foi importante, mas até 2011 (APC 28,4; p-valor 0,034), seguida de estabilidade nos segmentos posteriores (p-valor≥0,524). As taxas de Madureira (15ª) superaram às da área a partir de 2019, mas a tendência crescente só ocorreu até 2011, embora com significância estatística limítrofe (APC 24,0; p-valor 0,056). Na Pavuna (25ª) as taxas foram superiores às da área em 2007 e no período 2014-2017, mas com tendência crescente até 2016 (APC 12,0; p-valor 0,018). 

Na área de planejamento 5.1, as taxas superaram às do município a partir de 2010, mas com tendência de crescimento até 2012 (APC 38,5; p-valor 0,001). Na região de Bangu (17ª), as taxas foram superiores às da área, exceto em 2018, com tendência crescente também até 2012 (APC 39,3; p-valor 0,001). Em contrapartida, em Realengo (33ª), as taxas foram inferiores às da área, exceto em 2018, com tendência de aumento até 2011 (APC 41,5; p-valor 0,035). As taxas da área de planejamento 5.3 superaram as do município no período 2011-2015, mas com tendência crescente até 2011 (APC 41,5; p-valor 0,035).

Os dados sobre raça/cor da pele apresentaram alto percentual de ignorado na notificação de sífilis congênita no Sistema de Informação de Agravos de Notificação, com menor percentual em 2017 (24,2%) e maior em 2011 (48,2%). O Sistema de Informações sobre Nascidos Vivos apresentou alto percentual em 2011 (36,4%) e variou de 1,0% em 2013 a 3,5% em 2021.

As mães negras tinham proporcionalmente escolaridade e idade mais baixa (p-valor 0,004) do que as mães brancas (Tabela 1). Também apresentaram menor realização de pré-natal (p-valor<0,001) e tratamento para sífilis na gestação (p-valor<0,001) e do parceiro (p-valor 0,013). Destacou-se o alto percentual de casos cuja mãe realizou pré-natal, tanto para brancas quanto para negras, com diagnóstico realizado durante o pré-natal, mas com tratamento materno inadequado. 

As taxas entre mães brancas foram as mais baixas (Figura 4), com a maior taxa em 2014, mas mantendo-se estável no período (p-valor 0,647). As taxas entre as mães pardas foram mais altas do que entre as brancas, sendo maior em 2017; contudo, a queda observada na tendência não foi estatisticamente significativa (APC -1,9; p-valor 0,306). As taxas mais altas foram entre mães pretas, abaixo das pardas em 2017 e 2018, com tendência de queda no período (APC -6,1; p-valor 0,040).

**Tabela 1:** Características dos casos de sífilis congênita, segundo raça/cor da pele materna. Município do Rio de Janeiro, 2011-2021 (n=9.693)

Características	Branca	Negra	p-valor
	n (%)	n (%)	
Faixa etária materna (anos)			0,004
out/19	549 (24,4)	1.929 (26,6)	
20+	1.703 (75,6)	5.326 (73,4)	
Ignorado	41 (1,8)	145 (2,0)	-
Escolaridade materna			0,004
Até a 4ª série completa do ensino	172 (9,9)	672 (12,5)	
fundamental			
5ª série ou superior	1.566 (90,1)	4.711 (87,5)	
Ignorado	555 (24,2)	2.017 (27,3)	-
Realização de pré-natal			<0,001
Sim	2.020 (90,8)	6.171 (86,7)	
Não	204 (9,2)	950 (13,3)	
Ignorado	69 (3,0)	279 (3,8)	-
Momento do diagnóstico de sífilis materna			0,195
Durante o pré-natal	1.347 (61,0)	4.250 (59,5)	
No momento do parto/curetagem	787 (35,6)	2.602 (36,5)	
Após o parto	70 (3,2)	243 (3,4)	
Não realizado	6 (0,3)	43 (0,6)	
Ignorado	83 (3,6)	262 (3,5)	-
Esquema de tratamento materno			<0,001
Adequado	96 (4,7)	333 (5,1)	
Inadequado	1.398	4.110 (63,1)	
	-68,7		
Não realizado	541	2.072 (31,8)	
	-26,6		
Ignorado	258 (11,3)	885 (12,0)	-
Tratamento do parceiro			0,013
Sim	327 (23,0)	870 (19,9)	
Não	1.094 (77,0)	3.504 (80,1)	
Ignorado	872 (38,0)	3.026 (40,9)	-
Total	2.293 (100,0)	7.400 (100,0)	-

**Figura 4:** Taxas de incidência de sífilis congênita em menores de 1 ano de idade, segundo raça/cor da pele. Município do Rio de Janeiro, 2007-2021

## Discussão

A taxa de incidência de sífilis congênita em menores de 1 ano de idade apresentou crescimento expressivo de 2007 a 2012, com queda posterior até 2019, embora não estatisticamente significativo. A taxa ocorreu de forma heterogênea entre as áreas de planejamento e foi maior em crianças com mães pretas e pardas. 

A principal limitação do estudo foi a incompletude na variável raça/cor da pele na base de dados que inviabilizou maior compreensão da influência na tendência da taxa de sífilis congênita. Estudos realizados em Recife ^14^, em Belo Horizonte ^15^ e no município do Rio de Janeiro ^9^ identificaram que crianças cujas mães eram negras e com baixa escolaridade apresentavam risco acrescido para sífilis congênita. Outra limitação foram as flutuações das taxas, provavelmente aleatórias, em algumas áreas. Não foi utilizado o procedimento de médias móveis, pois a agregação em períodos poderia mascarar o impacto das políticas públicas. Utilizou-se o método de regressão *joinpoint* que realiza alisamento da reta para estimar a tendência por segmento. A subnotificação pode ter influenciado os resultados e está associada à unidade de atendimento e não às características individuais ^16^. Estudo que avaliou morte fetais e infantis por sífilis congênita chegou a identificar 0,9% de subnotificação no Sistema de Informação de Agravos de Notificação e 7,0% no Sistema de Informação sobre Mortalidade ^17^.

A queda da taxa de incidência da sífilis congênita coincidiu com a implantação da Rede Cegonha, política que apresentava a humanização na rede pública como foco do cuidado às gestantes, valorizando a importância de enfermeiras obstétricas e parteiras na assistência ^18^. A escassez de penicilina benzatina, antibiótico utilizado para o tratamento da sífilis em gestantes e seus conceptos, pode ter minimizado o impacto da queda das taxas de sífilis entre 2014 e 2017 ^19^. A partir de 2018, houve recrudescimento da doença, logo após o desmonte da Atenção Básica no município do Rio de Janeiro ^20^, sendo agravada com a pandemia de covid-19. O agravamento da sífilis congênita não foi estatisticamente significativo, provavelmente pelo pequeno tempo de seguimento. 

O efeito da implantação de política de valorização da assistência pré-natal foi lenta, com queda suave na tendência da sífilis congênita. Em contrapartida, o desmonte teve impacto imediato, expressando aumento exponencial da tendência. Tais hipóteses não puderam ser confirmadas estatisticamente, além de não terem sido observadas no país, nem nas macrorregiões, cuja tendência foi crescente de 2011 a 2021 ^21^. A expressiva redução de nascidos vivos a partir de 2018 não explicou o aumento observado da taxa de incidência de sífilis congênita, pois a tendência com o ajustamento das taxas, pela média de nascidos vivos no período, foi similar às taxas não ajustadas. 

Em 2022, houve a substituição da Rede Cegonha pela Rede de Atenção Materno e Infantil em todo o país ^22^ ^,^ ^23^. Isso pode agravar a assistência materna, pois a Rede de Atenção Materno e Infantil prioriza um modelo hospitalocêntrico e medicocêntrico em oposição ao cuidado territorializado conduzido na Estratégia Saúde da Família, além de estar desconectada com a realidade nacional e aos princípios do Sistema Único de Saúde ^24^. Em 2023, essa Rede foi invalidada ^25^ e o seu impacto ainda é desconhecido.

A taxa de incidência de sífilis congênita não foi homogênea no município. As áreas de planejamento 1.0, 3.3 e 5.1 apresentaram taxas maiores do que o município em todo o período. Na área 2.1, localizada na zona sul, apesar de taxas mais baixas, a tendência foi crescente, provavelmente influenciada por áreas altamente povoadas e mais carentes como a favela da Rocinha, com alta taxa de sífilis congênita, superando a do município. Situação similar foi observada na área 4, expressando o contraste das taxas mais baixas na Barra da Tijuca com as mais altas na favela Cidade de Deus. A favela do Jacarezinho também apresentou alta taxa de sífilis congênita no período, discrepando com as demais regiões da 3.2. Apenas em Guaratiba e no Complexo do Alemão não foram observadas recrudescimento das taxas a partir de 2018. As taxas do Complexo do Alemão ficaram abaixo do município e da sua área (3.1). Estudos em Recife ^14^ e no município do Rio de Janeiro ^9^ ^,^ ^10^ identificaram distribuição heterogênea entre os bairros, com maior taxa de sífilis congênita nos piores indicadores socioeconômicos e assistenciais. 

A oferta de penicilina foi heterogênea nas áreas de planejamento, com maior desabastecimento nas 3.0 e 5.0, e em 2014, 2015 e 2017 ^19^. Tal situação, contudo, não foi evidenciada na tendência da taxa de incidência de sífilis congênita nessas áreas. Apenas a 3.3 apresentou um recrudescimento da taxa a partir de 2013, mas com queda a partir de 2016, e não chegando a influenciar a mudança na tendência, pois já apresentava taxas altas e em ascensão desde 2007. 

A maioria das mães das crianças com sífilis congênita realizou e teve diagnóstico de sífilis no pré-natal, mas o tratamento foi inadequado ou não realizado - situação similar aos dados nacionais ^21^. Apesar dos esforços, a sífilis permanece como grave problema de saúde pública devido às falhas na assistência pré-natal ^26^. A alta rotatividade de profissionais na Atenção Básica, em função de contratos em serviços terceirizados, pode contribuir para esse quadro, visto que a substituição de profissionais geralmente não é acompanhada de capacitação, o que compromete a qualidade da assistência prestada.

Os dados sobre raça/cor da pele ignorados foram altos no Sistema de Informação de Agravos de Notificação - mesmo a partir de 2017, quando o preenchimento se tornou obrigatório em todos os sistemas de informação ^27^ - e apresentaram mais que o dobro dos percentuais observados no país em todo o período analisado ^21^. Quando os dados ignorados ou ausentes de uma variável excedem 10,0%, a análise é comprometida, pois tais dados podem não se distribuir aleatoriamente para as demais categorias ^28^. Diante dessa situação, a interpretação dos dados sobre raça/cor da pele deve ser realizada com cautela. 

Neste estudo, as mães negras apresentaram menor percentual de realização de pré-natal do que as brancas. As taxas de incidência de sífilis congênita para crianças com mães pretas e pardas foram maiores do que nas brancas, mas todas com tendência de queda até 2019 e recrudescimento posterior. A desigualdade em relação ao acesso pré-natal e à realização de exames segundo raça/cor da pele da gestante foi identificada anteriormente a partir da Pesquisa Nacional de Saúde ^29^ e da literatura mundial ^30^.

Apesar de o diagnóstico e o tratamento serem efetivos e de baixo custo, o esforço para o combate da sífilis congênita no município do Rio de Janeiro ainda não obteve o êxito desejado, em particular nos grupos mais vulneráveis, como a população negra e residentes em favela. O desmonte da Atenção Básica e a fragilidade dos vínculos contratuais são alguns dos empecilhos. A extinção da Rede de Atenção Materno e Infantil é um avanço ao retrocesso instalado, mas não é suficiente. 

Faz-se necessária a estruturação de política de recursos humanos para o Sistema Único de Saúde, em particular para a Atenção Básica, com a finalidade de garantir a fixação do profissional na rede e, consequentemente, menor custo em capacitação e maior resolutividade na assistência prestada. Ademais, é fundamental investir em capacitação para uma escuta qualificada, de forma a ampliar o vínculo e o desenvolvimento de pesquisas, com o objetivo de identificar estratégias diferenciadas para atender a população, visando à maior adesão ao tratamento da gestante e do parceiro.

## Data Availability

Conforme formulário sobre Conformidade com a Ciência Aberta, os conteúdos subjacentes ao texto do manuscrito estarão disponíveis após a publicação sob demanda aos autores.
